# Determining the distribution of granule diameter from biological sludge

**DOI:** 10.1016/j.mex.2018.06.002

**Published:** 2018-06-22

**Authors:** Inaê Alves, Valéria Del Nery, Eloisa Pozzi, Marcia Helena Rissato Zamariolli Damianovic, Eduardo Cleto Pires

**Affiliations:** Departamento de Hidráulica e Saneamento, Escola de Engenharia de São Carlos (EESC), Universidade de São Paulo (USP), Av. Trabalhador São-carlense, 400, São Carlos-SP, 13566-590, Brazil

**Keywords:** Granulometry assay, Granulometric biological sludge, Granules size

## Abstract

Anaerobic granule sizes from various types of anaerobic biological wastewater treatments were investigated in order to understand the influence of this characteristic on the performance of the treatment system. To date, there is no standardised methodology in the current literature, which provides details of a process to obtain data, such as a suitable sample volume, a description of the precision and limitations of the techniques used. Therefore, the aim of this protocol is to standardise the granulometry assay that can measure granule sizes accurately and quickly. In addition, the proposed methodology comprises about 1500–3000 granules in a single sample, a representative number compared to the currently applied methodologies.

## Highlights

• Simple and fast method for measurement of biological granule size distribution using standard scanners and image analysis software.
• Requires small samples and can provide the size of 1500–3000 granules approximately using a single sample.
• Process calibrated and validated using high quality ball bearing spheres.

## Graphical abstract

**Figure:**
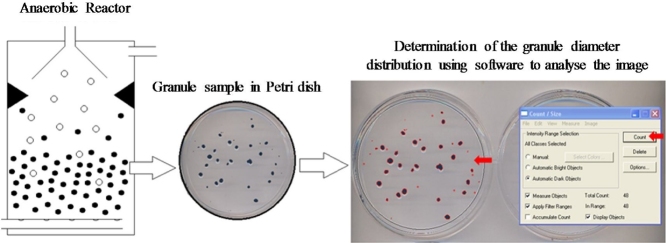

## Details of the method

The proposed protocol for determining the granules was based on the methodology used by Del Nery et al. [1]. In their work, Del Ney et al. [1] used a special apparatus dedicated to capture photo-macrographs that were used as inputs for granule size determination using an image analysis software, the Image Pro-Plus, with which one can perform diameter and area measurements, among other image analysis functions. With the development of high definition scanners the use of dedicated apparatus for image capture is no longer necessary, as the current work shows. Two tests were performed to evaluate the suitability of using scanners for the granulometry assay: a benchmark software test and a statistically representative sample volume evaluation, which was not evaluated by Del Nery et al. [1]. In order to determine the benchmark of the software, and the consequent precision of the granulometric measurement technique, a test using calibrated steel spheres (for granule simulation) was carried out. Three sample volumes – 5, 10 and 15 mL – were studied to evaluate the appropriate volume for the assay. After investigating the accuracy and precision of the software and the volume required for the sample, the methodology for collecting and preparing the granule sample was determined.

## Software and equipment used in this experimental procedure

- Image Pro-Plus Software (version 6.0);
- HP Scanjet scanner 3770;
- precision ruler;
- calibrated spheres of different diameters: 1, 2, 3, 4 and 5 mm;
- glue;
- volumetric flask;
- 6 Petri dishes;
- Plastic Pasteur pipette;
- water;
- MS Excel or similar program (for data acquisition and calculation).

N.B.: any scanner and image analysis software with similar characteristics can be used.

## Benchmarking the image analyser software

For initial calibration of the software, the following steps should be taken:

The image of a precisely marked ruler is captured; in this case a millimetric one using the scanner. It is important to check that the scanner does not introduce distortion into the image shape, i.e., the image of a square should be reproduced as a square.

1 Using the Image Pro-Plus software (version 6.0) opens the captured image of the ruler.
2 Select the options: “Measure” > “Calibration” > “Spatial”.
3 Select the options: “New” > change unit to millimeter > “Image”.
4 The length is marked and it informs the software of the millimeter value of the marked length. Here a 10 mm length is shown. The experimenter may want to try other lengths to improve the accuracy.
5 Give a name to the performed calibration or maintain the software option (Spatial Cal 0) > “Apply”.

The reader may refer to the Supplementary material which contains figures showing captures of the software screen.

## Evaluating of confidence level that the Image Pro-Plus software, version 6.0, presents

Tests were performed to evaluate the level of confidence that the program (Image Pro-Plus software, version 6.0) presents. Basically, calibrated steel spheres, i.e. of a known size were used to simulate the granules. The diameter determination protocol described herein was followed:

1 The spheres were placed in Petri dishes (as they were perfect spheres, they had to be glued to keep them in place in the Petri dishes);
2 The image of the spheres was captured using the scanner and the diameters were determined using the Image Pro-Plus software (version 6.0);
3 The diameter provided for the calibrated spheres was compared, establishing the accuracy and precision of the software in providing the dimensions of the analysed images. The most important characteristic is precision as accuracy can be adjusted by calibration.

**Fig. 1:**
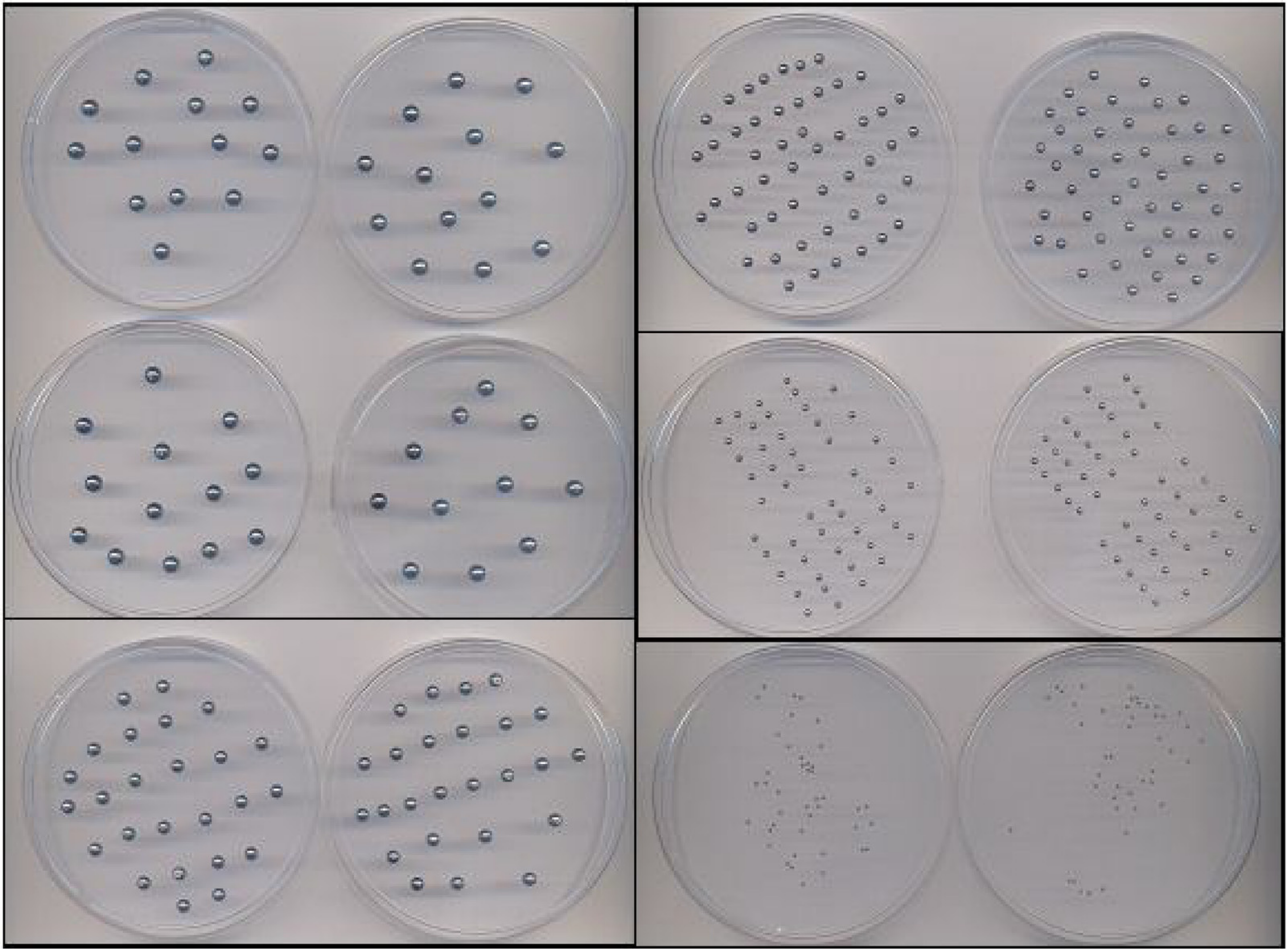
Calibrated steel spheres used to verify the precision of software.

The first assay consisted of analysing spheres of the same diameter separately and the second comprised spheres of different diameters in the same image (Table 1, and Fig. 1). The mean of the diameters and the standard deviation indicated small deviations between the actual values and those observed (Table 2). The error indicates the accuracy and the standard deviation and variation coefficient indicate the precision of the measurement.

**Table 1:** Calibration tests of Image Pro-Plus software (version 6.0): number of spheres and Petri dishes used to evaluate the image analysis software.

1st Assay	Sphere diameter	Nº of spheres	N° of used Petri dishes
Test 1	5 mm	50	4
Test 2	4 mm	50	2
Test 3	3 mm	100	2
Test 4	2 mm	100	2
Test 5	1 mm	100	2

2nd Assay	Varied diameters	Nº of spheres	N° of used Petri dishes
Test 6	5 mm	80	2
	4 mm		
	3 mm		
	2 mm		
	1 mm

**Table 2:** Calibration tests of Image Pro-Plus software (version 6.0): measurement results.

Actual diameter of the spheres (mm)	Mean diameter obtained (mm)	Error (mm)	Standard deviation (mm)	Coefficient of variation (%)
1.00	0.92	0.08	0.04	4.4
2.00	1.86	0.14	0.05	2.7
3.00	2.91	0.09	0.08	2.7
4.00	3.86	0.14	0.08	2.1
5.00	4.93	0.07	0.07	1.4

It can be observed that in this case the error is always negative, close to 0.1 mm (Table 2) and can be considered a systematic error, thus a simple calibration curve could be used to obtain the correct size of the spheres of an unknown diameter. More importantly for the measurement procedure is to observe that the standard deviation and variation coefficient indicate that the experimental precision is sufficient for biological granule size determination. Granules are not perfect spheres because they have uneven surfaces. Therefore, measuring the size of the granules is actually an approximation. The software calculates the average length of diameters measured at 2° intervals passing through the centroid of the object (granule). Thus, the error observed in the software response can be considered negligible.

**Fig. 2:**
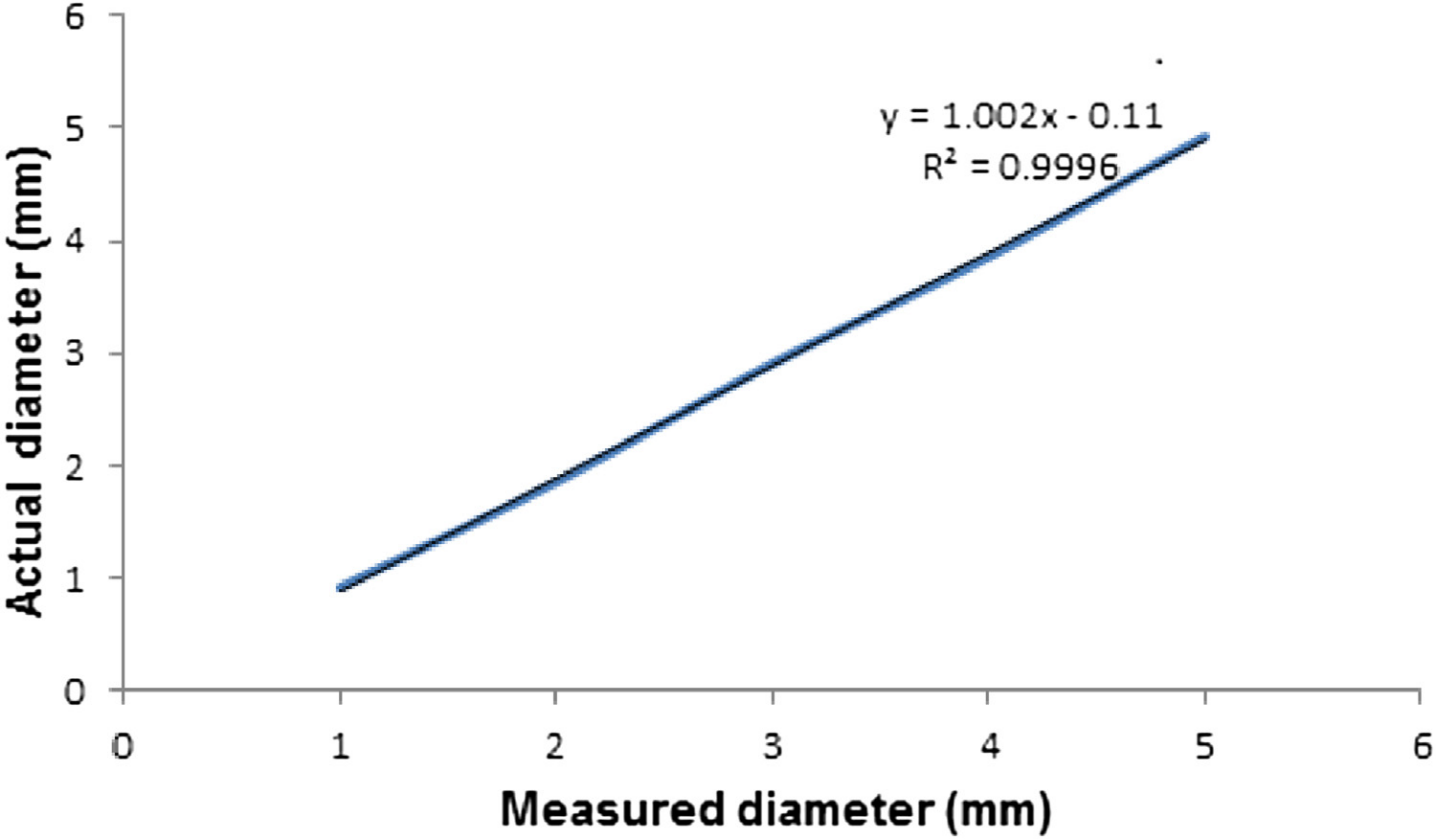
Relation between the actual and measured diameter and its R².

The value of R², which varies between 0 and 1, was 0.999 (Fig. 2), reinforcing the validity of the diameter measurement procedure using a scanner image and its processing in the chosen software (Image Pro-Plus).

## Procedure for measuring the granule size

1 Using the Image Pro-Plus software (version 6.0) opens the first captured image to be analysed;
2 Select “Measure” > “Calibration” > “Select Spatial” >. Select the “Spatial Cal 0” calibration option (this selection must be performed whenever a new image is opened for analysis) > “Apply”;
3 Select “Ellipse” > mark the Petri dish in the image (do this carefully so as not to select the edge of the dish);
4 Select “Measure” > “Count/Size”;
5 Select “Measurements”> “Select Measurements”;
6 Select the “Diameter Mean” function (or/and another function that you are interested in) > “OK”;
7 Select “Count” (check if the dark objects of interest, the granules, were marked in red by the software);
8 Select “File”> “Clipboard”;
9 Transfer the values copied to an Excel page for later statistical data analysis;
10 Repeat the procedure for each Petri dish in the image (the circumference obtained by the function “Ellipse” can be dragged with the mouse);
11 Repeat the procedure for the other images (as already mentioned, the initial calibration “Spatial Cal 0” has to be selected, whenever a new image is opened).

The reader may refer to the Supplementary material which contains figures showing captures of the software screen.

## Collecting granule samples for granulometric determination

It is important that the sample is representative. For example, if the objective is to evaluate the sludge blanket of a UASB (Upflow Anaerobic Sludge Blanket) reactor, the sample should be drawn from several points, and in the same amount, along the reaction profile of the reactor so that, in fact, the sample represents the granules of the whole sludge blanket. It is important to follow sampling order as the granule must be collected from the highest point to the lowest of the reaction zone due to the rearrangement of the granules in the system when a sample is taken from the reactor. This is specially an important factor in smaller reactors, as bench scale and pilot scale reactors. In pilot or real scale reactors with a larger volume of granular sludge, a composite sample of 1 L in total can be collected, taking approximately 100 to 200 mL from each point along the reaction zone, depending on how many sampling ports there are available. The sample volume for the particle size analysis is taken from the composite sample. If the objective is to analyse the sludge blanket granules at different points in the reactor, then the collection should be exact (e.g.: base, middle and highest point of the reactor sludge blanket).

## Preparing the granule samples for granulometric determination

The granular sludge sample is mixed (suitable sample volume is discussed in "Determining sample volume to measure granule dimensions") in approximately 1 L of water in a beaker. After mixing, it is necessary to wait for sedimentation of the granules (1–2 min) to discard the supernatant (between 700 and 800 ml). This process is repeated three times. At this stage, called the washing step, very fine granules (<0.4 mm) and no sedimentation property are discarded.

## Determining the sample volume to measure the size of the granules

Granule samples from a UASB reactor, used to treat a poultry slaughterhouse waste, previously described by Del Nery et al. [1], were utilised to determine the sample volume indicated for measuring the granule diameter.

This step consisted of adopting the methodology proposed by Del Nery et al. [1], who only used 10 mL of the sample for the granulometry assay. The appropriate volume was evaluated using 5, 10 and 15 mL of the whole granule sample to identify statistically which of the volumes presented the most significant response. The assay was done in triplicate.

**Fig. 3:**
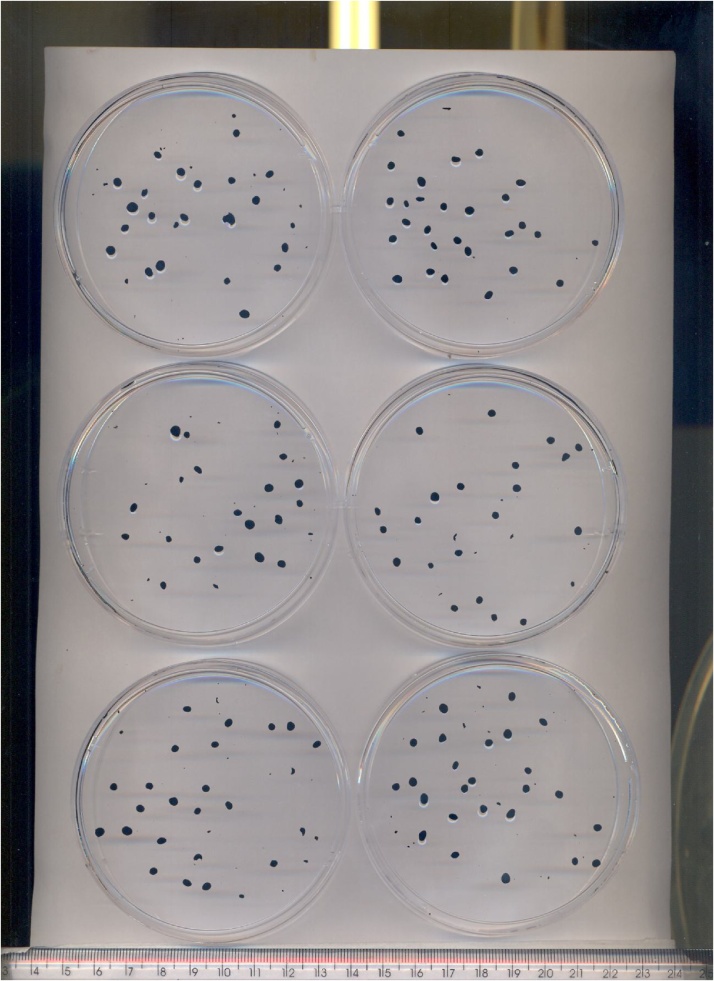
Granules exposed in Petri dishes for image analysis.

The samples went through the washing step described in the section “Preparing the granule samples for granulometric determination”. The size of the granules was identified in two steps: the images of the Petri dishes containing the granules were captured first (Fig. 3); then the dimensions were identified by image analysis using the Image Pro-Plus software (version 6.0), as described in the section on “Granulometric determination assay”.

**Fig. 4:**
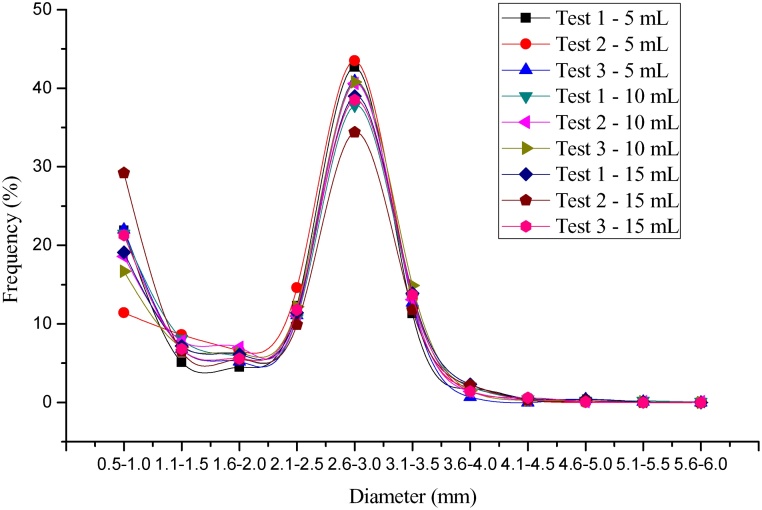
Test results to determine the sample volume for granulometry assay.

**Fig. 5:**
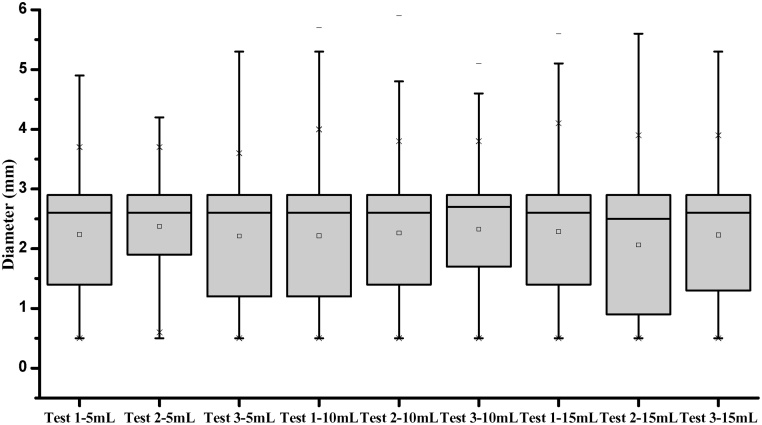
Test results to determine the sample volume for granulometry assay.

The assay performed with a volume of 10 mL of sample had a lower average of the standard deviation, variance and coefficient of variation (0.7, 1 and 3.5, respectively), considering all the established diameter ranges compared to the other tests with a volume of 5 and 15 mL (Fig. 4, Fig. 5, Table 3, Table 4, Table 5). However, the volume of 5 mL can also be used if the amount of sludge available is small, as is the case of bench scale reactors.

**Table 3:** Test results to determine the sample volume for granulometry assay.

Diameter	Frequency (%)								
	5 mL of sample			10 mL of sample			15 mL of sample		
	Test 1	Test 2	Test 3	Test 1	Test 2	Test 3	Test 1	Test 2	Test 3
0.5–1.0	21.9	11.4	22.0	21.5	18.6	16.7	19.1	29.2	21.3
1.1–1.5	5.1	8.6	6.8	8.1	7.8	7.0	7.2	6.5	6.8
1.6–2.0	4.5	6.6	5.2	5.8	7.0	6.3	6.1	5.5	5.6
2.1–2.5	12.3	14.6	11.1	10.7	11.0	12.2	11.4	9.9	11.8
2.6–3.0	42.7	43.5	40.9	37.8	40.6	40.8	39.0	34.4	38.5
3.1–3.5	11.3	13.2	12.7	13.3	13.1	14.9	13.9	11.8	13.7
3.6–4.0	1.8	1.9	0.7	2.0	1.4	1.5	2.3	2.2	1.4
4.1–4.5	0.4	0.3	0.0	0.4	0.3	0.3	0.5	0.3	0.6
4.6–5.0	0.1	0.0	0.4	0.2	0.1	0.2	0.4	0.2	0.1
5.1–5.5	0.0	0.0	0.1	0.2	0.1	0.1	0.1	0.0	0.0
5.6–6.0	0.0	0.0	0.0	0.1	0.1	0.0	0.0	0.0	0.0

**Table 4:** Descriptive statistics of the test results to determine the sample volume for the particle size assay.

	Mean			Standard deviation		
	5 mL of sample	10 mL of sample	15 mL of sample	5 mL of sample	10 mL of sample	15 mL of sample
	21.9	18.6	21.3	6.1	2.4	5.3
	6.8	7.8	6.8	1.7	0.6	0.3
	5.2	6.3	5.6	1.1	0.6	0.3
	12.3	11.0	11.4	1.8	0.8	1.0
	42.7	40.6	38.5	1.3	1.7	2.5
	12.7	13.3	13.7	1.0	1.0	1.2
	1.8	1.5	2.2	0.7	0.3	0.5
	0.3	0.3	0.5	0.2	0.0	0.1
	0.1	0.2	0.2	0.2	0.1	0.1
	0.0	0.1	0.0	0.1	0.1	0.1
	0.0	0.1	0.0	0.0	0.1	0.0
Total	100.0	100.0	100.0	1.3	0.7	1.0

**Table 5:** Descriptive statistics of the test results to determine the sample volume for the particle size assay.

	Variance			Coefficient of variation		
	5 mL of sample	10 mL of sample	15 mL of sample	5 mL of sample	10 mL of sample	15 mL of sample
	36.9	6.0	28.4	0.3	0.1	0.2
	3.0	0.3	0.1	0.3	0.1	0.1
	1.1	0.4	0.1	0.2	0.1	0.1
	3.2	0.7	1.0	0.1	0.1	0.1
	1.8	2.9	6.4	0.0	0.0	0.1
	1.0	1.0	1.4	0.1	0.1	0.1
	0.4	0.1	0.2	0.4	0.2	0.2
	0.0	0.0	0.0	0.7	0.2	0.3
	0.0	0.0	0.0	1.7	0.4	0.7
	0.0	0.0	0.0	a	1.1	1.5
	0.0	0.0	0.0	a	1.1	0.6
Total	4.3	1.0	3.4	3.8	3.5	3.9

a not possible to calculate because the sample mean is zero.

## Granulometric determination assay

The granulometric determination assay takes place in two steps: capturing and image analysis. In the first, the granules that sedimented in the washing step are placed in Petri dishes, paying attention to keeping them separate. It is of the utmost importance that the granules are separated carefully from one another because the image analyser program interprets the dark objects. Therefore, if two granules are together, the program recognises them as just one object. In addition, the granules must be handled with care to avoid damage that modifies their structure. Thus, in order to separate them, a tool that does not damage the sample should be used. The one used in this study was a plastic Pasteur pipette. The Petri dishes are positioned on a conventional scanner and then the image is obtained and saved. Each image can contain up to 6 Petri dishes (the maximum amount the scanner can hold), thus reducing the analysis time (Fig. 3).

In the image analysis step, the initial calibration of the software is carried out, then the analysis of the captured images described in the section “Procedure for measuring the granule size” is initiated.

## Additional information

The success of using UASB technology to treat a wide range of wastewater is strongly related to the anaerobic sludge granulation phenomenon. The characteristics of the granule in the sludge blanket are associated with numerous factors related to the nature of the wastewater and the design and operational characteristics of the reactor as pointed out by Grotenhuis et al. [2] and Ghangrekar et al. [3]. The cell grouping process, which begins when microorganisms become sensitive to environmental parameters or to the stress condition, comprises an important survival strategy because the cells protect themselves against external aggression. Adequate immobilisation of microorganisms is what will make the difference between a successful high rate treatment system and the others, regardless of the treatment system considered. Biomass immobilisation is a complete and stable metabolic arrangement that enables optimal environmental conditions for all its members, MacLeod, Guiot and Costerton [4].

The granulation phenomenon occurs continuously in UASB reactors and the increase or reduction of the granules size occurs according to the conditions imposed on the operating units. The monitoring of granular sludge size dynamics is a powerful tool in predicting reactor stability which associated with other monitoring parameters can clarify possible causes of UASB process imbalance during different operational periods as indicated by Puñal and Chamy [5] and Lettinga et al. [6].

One of the important characteristics of the granules in the sludge blanket is the high sedimentation velocity, which are beneficial for operating anaerobic reactors because there is no wash-out of viable biomass, Lu et al. [7]. Usually the granules range from 0.5 to 5 mm in diameter, which due to their sedimentation property, resist wash-out of the reactor even at high hydraulic loads Show et al. [8]. However, some researchers pointed out smaller bands of granules: Lu et al. [9] reported initial granular sludge consisting of well-settled black granules with about 80% showing the size from 0.4 to 4.0 mm in diameter, Del Nery et al. [1] observed smaller bands ranging from 0.1 to 3.5 mm and Gagliano et al. [10] reported a band of 0.5 to 3 mm as being the most frequent in these systems. Although there is increasing interest in understanding the granulation process and maintenance of the sludge blanket, there are no reports in the literature in which the methodology of granulometric determination of sludge is reported in detail, whereby a significant number of granules is analysed relatively quickly and simple.
