# Supplementary material for: Determining the distribution of granule diameter from biological sludge
Source: MethodsX. 2018 Jun 22;5:727–36. doi: 10.1016/j.mex.2018.06.002 (PMC6070658; doi:10.1016/j.mex.2018.06.002)
Supplement: Supplementary file 1 [file mmc1.docx]

**DETERMINING THE DISTRIBUTION OF GRANULE DIAMETER FROM BIOLOGICAL SLUDGE**

**Supplementary Material**

**Captures of the software screen: Initial calibration step.**


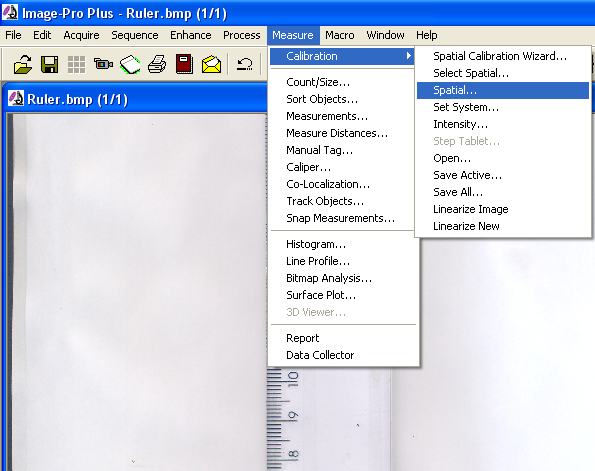


Figure 1. Step 1 for initial calibration of Image Pro-Plus software (version 6.0)


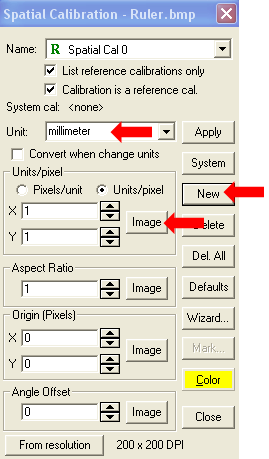


Figure 2. Step 2 for initial calibration of Image Pro-Plus software (version 6.0)


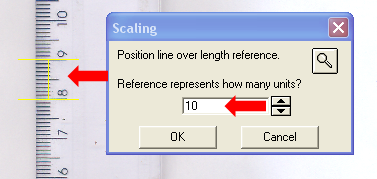


Figure 3. Step 3 for initial calibration of Image Pro-Plus software (version 6.0)


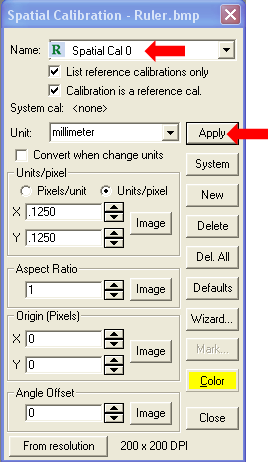


Figure 4. Step 4 for initial calibration of Image Pro-Plus software (version 6.0)

**Captures of the software screen: Granule size measurement step.**


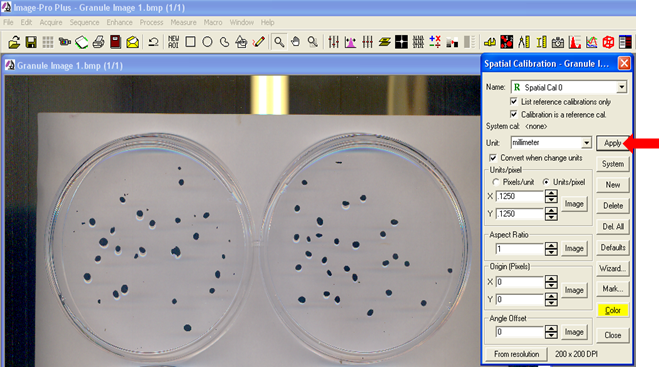


Figure5. Step 1 for image analysis using Image Pro-Plus software (version 6.0)


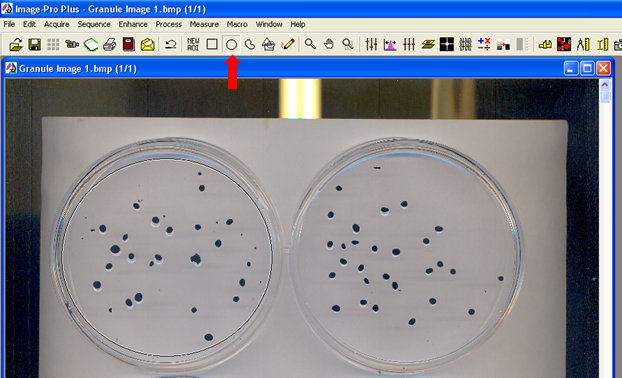


Figure 6. Step 2 for image analysis using Image Pro-Plus software (version 6.0)


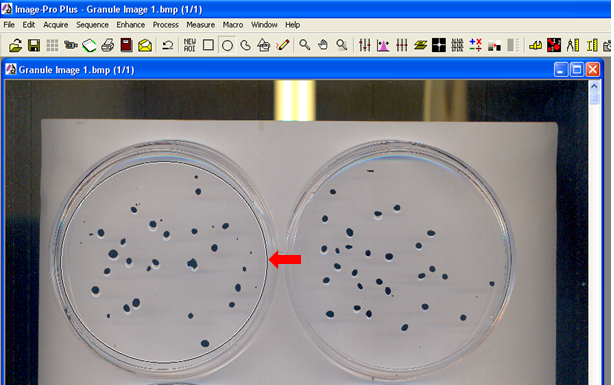


Figure 7. Step 3 for image analysis using Image Pro-Plus software (version 6.0)


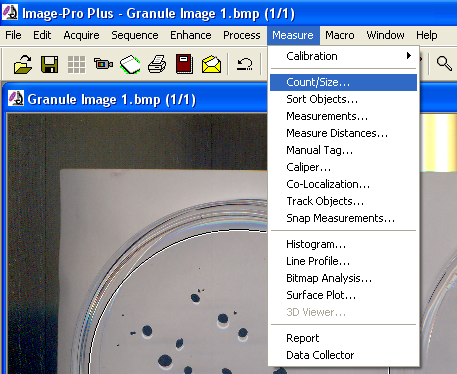


Figure 8. Step 4 for image analysis using Image Pro-Plus software (version 6.0)


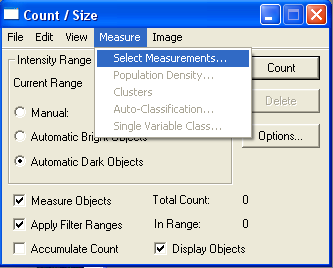


Figure 9. Step 5 for image analysis using Image Pro-Plus software (version 6.0)


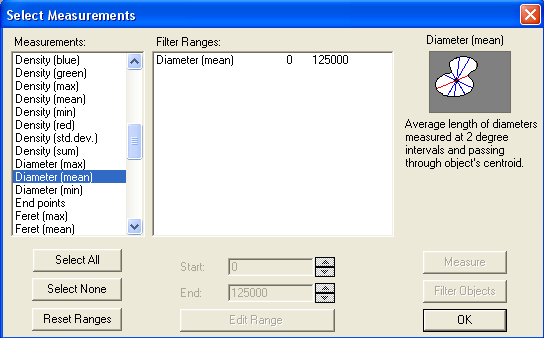


Figure 10. Step 6 for image analysis using Image Pro-Plus software (version 6.0)


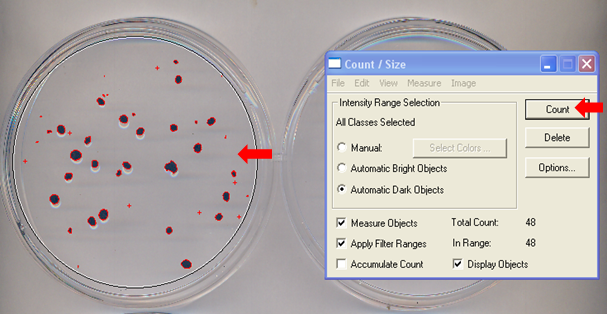


Figure 11. Step 7 for image analysis using Image Pro-Plus software (version 6.0)


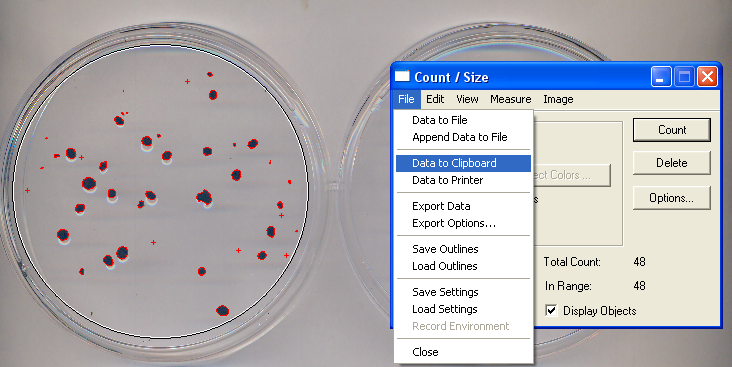


Figure 12. Step 8 for image analysis using Image Pro-Plus software (version 6.0)
