# Supplementary material for: Determining the distribution of granule diameter from biological sludge
Source: MethodsX. 2018 Jun 22;5:727–36. doi: 10.1016/j.mex.2018.06.002 (PMC6070658; doi:10.1016/j.mex.2018.06.002)
Supplement: Supplementary file 2 [file mmc2.docx]

**Method Article**

*** Title: DETERMINING THE DISTRIBUTION OF GRANULE DIAMETER FROM BIOLOGICAL SLUDGE**

***Authors:** Inaê Alves¹ (Corresponding author); Valéria Del Nery¹ ; Eloisa Pozzi¹ ; Marcia Helena Rissato Zamariolli Damianovic¹ ; Eduardo Cleto Pires¹

***Affiliations:** ¹Departamento de Hidráulica e Saneamento, Escola de Engenharia de São Carlos (EESC), Universidade de São Paulo (USP)

Av. Trabalhador São-carlense, 400, São Carlos-SP, 13566-590, Brazil.

***Contact email:** Inaê Alves¹ (Corresponding author) (E-mail: [inaealves89@hotmail.com](mailto:inaealves89@hotmail.com), [inaealves2207@gmail.com](mailto:inaealves2207@gmail.com))

Valéria Del Nery¹  (E-mail: [vdelnery@terra.com.br](mailto:vdelnery@terra.com.br))

Eloisa Pozzi¹ (E-mail: [elopozzi@sc.usp.br](mailto:elopozzi@sc.usp.br))

Marcia Helena Rissato Zamariolli Damianovic¹ (E-mail: [mzamariollidamianovic@gmail.com](mailto:mzamariollidamianovic@gmail.com))

Eduardo Cleto Pires¹ ([ecpires@sc.usp.br](mailto:ecpires@sc.usp.br))

*** Keywords:** Granulometry assay; Granulometric biological sludge; Granules size

**ABSTRACT**

***Abstract:** Anaerobic granule sizes from various types of anaerobic biological wastewater treatments were investigated in order to understand the influence of this characteristic on the performance of the treatment system. To date, there is no standardised methodology in the current literature, which provides details of a process to obtain data, such as a suitable sample volume, a description of the precision and limitations of the techniques used. Therefore, the aim of this protocol is to standardise the granulometry assay that can measure granule sizes accurately and quickly. In addition, the proposed methodology comprises about 1500–3000 granules in a single sample, a representative number compared to the currently applied methodologies.

**Highlights**

• Simple and fast method for measurement of biological granule size distribution using standard scanners and image analysis software.

• Requires small samples and can provide the size of 1500–3000 granules approximately using a single sample.

• Process calibrated and validated using high quality ball bearing spheres.

**SPECIFICATIONS TABLE**

| **Subject Area** | *• Engineering* |
| --- | --- |
| **More specific subject area:** | Sanitation |
| **Method name:** | DETERMINING THE DISTRIBUTION OF GRANULE DIAMETER |
| **Name and reference of original method** | Name: size of the granules  Reference: V. Del Nery, E. Pozzi, M.H.R.Z. Damianovic, M.R. Domingues, M. Zaiat, Granules characteristics in the vertical profile of a full-scale upflow anaerobic sludge blanket reactor treating poultry slaughterhouse wastewater, Bioresour. Technol. 99 (2008) 2018–2024. doi:10.1016/j.biortech.2007.03.019. |
| **Resource availability** |  |

***Method details:** The proposed protocol for determining the granules was based on the methodology used by Del Nery et al. [1]. In their work, Del Ney et al. [1] used a special apparatus dedicated to capture photo-macrographs that were used as inputs for granule size determination using an image analysis software, the Image Pro-Plus, with which one can perform diameter and area measurements, among other image analysis functions. With the development of high definition scanners the use of dedicated apparatus for image capture is no longer necessary, as the current work shows. Two tests were performed to evaluate the suitability of using scanners for the granulometry assay: a benchmark software test and a statistically representative sample volume evaluation, which was not evaluated by Del Nery et al. [1]. In order to determine the benchmark of the software, and the consequent precision of the granulometric measurement technique, a test using calibrated steel spheres (for granule simulation) was carried out. Three sample volumes – 5, 10 and 15 mL – were studied to evaluate the appropriate volume for the assay. After investigating the accuracy and precision of the software and the volume required for the sample, the methodology for collecting and preparing the granule sample was determined.

**Acknowledgements:** The authors acknowledge the National Council for Scientific and Technological Development (CNPq - Conselho Nacional de Desenvolvimento Científico e Tecnológico, Brazil), 142211/2015-0 and 304394/2009-2, and the São Paulo Research Foundation (Fapesp - Fundação de Amparo à Pesquisa do Estado de São Paulo, Brazil), 2013/17591-1 for supporting this study.

**Supplementary material *and/or* Additional information:** Material is in the other Attached

***References:** *[Include at least one reference, to the original publication of the method you customized.]*

[1] V. Del Nery, E. Pozzi, M.H.R.Z. Damianovic, M.R. Domingues and M. Zaiat, Granules characteristics in the vertical profile of a full-scale upflow anaerobic sludge blanket reactor treating poultry slaughterhouse wastewater, Bioresour. Technol. 99, 2008, 2018–2024, https://doi.org/10.1016/j.biortech.2007.03.019.

[2] J.T.C. Grotenhuis, J.C. Kissel, C.M. Plugoe, a J.M. Stams and a J.B. Zehnder, Role of substrate concentration in particle size distribution of methanogenic granular sludge in Uasb reactors, Water Res. 25, 1991, 21–27, https://doi.org/10.1016/0043-1354(91)90094-7.

[3] M.M. Ghangrekar, S.R. Asolekar, K.R. Ranganathan and S.G. Joshi, Experience with UASB reactor start-up under different operating conditions, Water Sci. Technol. 1996, 421–428, https://doi.org/10.1016/0273- 1223(96)00674-9.

[4] Fa. MacLeod, S.R. Guiot and J.W. Costerton, Layered structure of bacterial aggregates produced in an upflow anaerobic sludge bed and filter reactor, Appl. Environ. Microbiol. 56, 1990, 1598–1607.

[5] A. Puñal and R. Chamy, Dynamics of Granules Physical Properties in UASB and EGSB Reactors for the Treatment of Medium and Low Concentrated Wastewasters, 2004, 1139–1144.

[6] G. Lettinga, J. Field, J.B. van Lier, G. Zeeman and L.W.H. Pol, Advanced anaerobic wastewater treatment in the near future, Water Sci. Technol. 35, 1997, 5–12, <https://doi.org/10.1016/S0273-1223(97)00222-9>.

[7] Y. Lu, F. Slater, R. Bello-Mendoza and D.J. Batstone, Shearing of biofilms enables selective layer based microbial sampling and analysis, Biotechnol. Bioeng. 110, 2013, 2600–2605, <https://doi.org/10.1002/bit.24947>.

[8] K.Y. Show, Y. Wang, S.F. Foong and J.H. Tay, Accelerated start-up and enhanced granulation in upflow anaerobic sludge blanket reactors, Water Res. 38, 2004, 2292–2303, <https://doi.org/10.1016/j.watres.2004.01.039>.

[9] X. Lu, G. Zhen, A.L. Estrada, M. Chen, J. Ni, T. Hojo, K. Kubota and Y.Y. Li, Operation performance and granule characterization of upflow anaerobic sludge blanket (UASB) reactor treating wastewater with starch as the sole carbon source, Bioresour. Technol. 180, 2015, 264–273, <https://doi.org/10.1016/j.biortech.2015.01.010>.

[10] M.C. Gagliano, S.B. Ismail, A.J.M. Stams, C.M. Plugge, H. Temmink and J.B. Van Lier, Bio fi lm formation and granule properties in anaerobic digestion at high salinity, Water Res. 121, 2017, 61–71, https://doi.org/10.1016/j.watres.2017.05.016.
